# A Strain-Based Method to Estimate Tire Parameters for Intelligent Tires under Complex Maneuvering Operations

**DOI:** 10.3390/s19132973

**Published:** 2019-07-05

**Authors:** Mª Fernanda Mendoza-Petit, Daniel Garcia-Pozuelo, Vicente Diaz, Oluremi Olatunbosun

**Affiliations:** 1 Mechanical Engineering Department, Universidad Carlos III de Madrid, Avd. De la Universidad, 28911 Madrid, Spain; 2 School of Mechanical Engineering, University of Birmingham, Edgbaston B15 2TT, UK

**Keywords:** tire-road forces estimation, slip angle estimation, gauge sensors, fuzzy logic system, load transfer estimation, simulation results, normalization, lateral force empirical model

## Abstract

The possibility of using tires as active sensors opens the door to a huge number of different ways to accomplish this goal. In this case, based on a tire equipped with strain sensors, also known as an Intelligent Tire, relevant vehicle dynamics information can be provided. The purpose of this research is to improve the strain-based methodology for Intelligent Tires to estimate all tire forces, based only on deformations measured in the contact patch. Firstly, through an indoor test rig data, an algorithm has been developed to pick out the relevant features of strain data and correlate them with tire parameters. This information of the tire contact patch is then transmitted to a fuzzy logic system to estimate the tire parameters. To evaluate the reliability of the proposed estimator, the well-known simulation software CarSim has been used to back up the estimation results. The software CarSim has been used to provide the vehicle parameters in complex maneuvers. Finally, the estimations have been checked with the simulation results. This approach has enabled the behaviour of the intelligent tire to be tested for different maneuvers and velocities, providing key information about the tire parameters directly from the only contact that exists between the vehicle and the road.

## 1. Introduction

Nowadays, the tire is largely regarded as a passive element in the automotive field, even though many studies have pointed out the importance of the tire in the dynamic behaviour of the vehicle. In spite of the interesting developments in intelligent tires along the last years, one of the greatest achievements of the intelligent tire systems commercialized to date is the Tire Pressure Monitoring System (TPMS). This is a proof of the difficulty for a more ambitious intelligent tire concept to meet all the requirements in commercial tires. Overall, the number of sensors in a vehicle continues to increase. Many of these sensors provide vital information such as longitudinal and lateral acceleration, yaw rate, engine torque, etc. Nonetheless, very few sensors are able to provide accurate information related to tire-road interaction parameters. 

Safety is the foremost reason to develop an intelligent tire as an active sensor able to provide useful information which is otherwise hard to measure, e.g., load transfer, tangential forces, tire conditions, road conditions or friction coefficients. Such information would enhance the functionality of different control systems such as Anti-Lock Braking Systems (ABS), Traction Control Systems (TCS), Electronic Stability Control (ESC), and Suspension Control Systems (SCS). Matsuzaki and Todoroki [1] suggested the possibility of developing an optimized braking control and road condition warning system using a strain-based system, where the road condition warning would be actuated if the recorded friction coefficient at certain slip ratio is lower than a safe reference value. Using Intelligent Tires to complement the current control systems may help prevent losing control of the vehicle in adverse road conditions.

There is a large number of interesting published works about tire parameter estimation. An example is, the neural model based on recursive lazy learning to predict the tire characteristics. However, adjustment of the model is required each time new data is presented [2]. Doumiati et al. [3,4], implemented virtual sensors to estimate the lateral tire force and sideslip angle, from a simplified four-wheel vehicle model. Despite the fact the experimental results show the potential of the estimation method, it needs to measure the data from the on-board vehicle sensors to estimate the tire parameters. According to Lee et al. [5], observers estimation provides, under the severe driving condition accompanied with large combined slips and abrupt wheel load changes, unreliable values and also, considerable uncertainties can be accumulated during the estimation process, which can be due to the use of a simple vehicle model. One of the most used is the Magic Formula [6,7,8], nonetheless, according to Rajamani [9], the results from analytical elastic foundation models (the brush models) can match the data very well for cases of pure lateral or pure longitudinal force generation. However, the analytical models do not always lead to quantitatively accurate results. Differences from experimental data are observed, especially at large slip and at combined slip. 

Other instrumented equipment, e.g. dynamometric hubs and dynamometric plates, allow measuring tire forces, but they are usually expensive and not easy to use in conventional vehicles. 

Due to the influence of tire forces and the slip angle on the dynamic control of the vehicle, it is important to estimate reliable results. Following different approaches and transducers many prototypes of instrumented tires have been produced to develop the concept, complementing the current vehicle control systems. The results of these studies in terms of the achieved reliability are diverse, but the main goal of the sensors used into the intelligent tires is to characterize the contact patch in all cases. For this reason, these sensors are usually located as near as possible to the contact patch and present advantages and disadvantages as it is explained in next analysis.

Accelerometers based on micromechanical systems (MEMS) are widely used. This type of sensor assures signal linearity and stability over time and insensitivity to temperature change, however, it is very sensitive to the noise (high frequency vibration) generated when the tire rolls on the road. Hence, it is difficult to extract the characteristics of interest without advanced signal processing [5]. On the other hand, the high vibration levels inside a tire have the potential to generate electrical power using vibration based energy harvesting techniques. As result, it has been proposed a piezoelectric energy harvesting system with energy storage as intelligent tire sensor based on piezoelectric transducers (accelerometers). The onboard vibration energy harvesting unit has been adapted to the tire vibration spectra and the superimposed acceleration signal [10,11]. To optimize the harvester performance with changing dominant tire vibration frequencies, there were used an artificial neural network (ANN) trained at different tire operating conditions to ensure broadband operation of the harvester [12]. 

Other types of sensors used in the intelligent tire field are based on the deformation of the tire as results of the contact surface interaction are introduced. Xiong and Tuononnen [13], explained that the deformation of parts of a tire is the direct result of tire–road interactions. In this study, a flexible ring tire model and a laser sensor were used to analyze the in-plane deformations of the tire carcass. It was found that the radial deformation of the tire carcass provides information on both the longitudinal and vertical forces acting on the tire, being mainly attributed to the coupled effect between the in-plane tire deformations caused by the inextensibility of the tire carcass in a modern radial tire.

Roveri et al. [14] proposed a device consisting of Fiber Bragg Grating (FBG) sensors and a light spectrum analyzer (optical strain measurements in rolling tires). This device allows the acquisition of the tire strain along an array of measurement points. Based on experimental and theoretical methodologies, it can set the pillars for a new system for real-time identification of the tire stress during rolling and the residual grip estimation.

Different studies have been conducted in intelligent tires based on strain gauges. One of the first issues with this sort of sensors is their location. Cheli et al [15] studied the influence of the strain gauge position on the wheel rim on a measurement system. It was concluded that the results were apparently independent of the position of the strain gauge. Some other researches have successfully developed intelligent tires based on strain gauge measurements, achieving advanced systems to estimate tire characteristics [16,17,18,19,20]. Matsuzaki and Todoroki [1] investigated the relationship between variation in strain on the inner surface of pneumatic tires and tire mechanical parameters by carrying out finite element analysis and simulating the strain sensor signal when a tire rotates. In general, it has been found that the strain sensors measurements can be directly related to tire deflection and operational conditions [5] with a little margin for error under dynamic conditions [19,20]. In most cases, they are attached to the inner surface of the tire to contribute to a lower probability of damage.

Other studies with strain gauges as sensors have been fitted for real-time conditions. This is the case of Garcia-Pozuelo et al. [21,22]. In this document a real-time physical model suitable describe the dynamics of intelligent tires based on measurements of strains grounded on a flexible ring on a viscoelastic foundation. It can reproduce the tire longitudinal dynamics for both concentrated and distributed forces by a discrete approach. The solution of the model dynamics has been obtained in the closed form and the model parameters have been identified from experimental data. This model could be used for predicting the dynamical behavior of both the strain-based intelligent tire sensor and the laser based one. It also may be used in the design of real-time observers for tire condition based on tire strain measurements. 

Yunta et al. [23] prove the camber angle influence on the strain signal. In an indoor tire test rig experimental tests were carried out to measure the tire tread deformation by means of strain gauges under different working conditions, such as vertical load, slip angle, camber angle. This study shows in a very clear way the influence of the strain gauges location on the results.

These studies evidence that the strain sensors meet the requirements to estimate the tire parameters regarding the working condition and tire properties; as well as the reliability, repeatability and easily installed [16,17,19,20] without adding weight to the measured surface (which can represent adding stress to the surface). The achievement of an intelligent tire system is a concept that is being currently developed and presents many future ways and possibilities. In this manuscript some improvements over the strain-based intelligent tire are proposed, providing new information for driving behavior and warnings about slippery road by measuring the potential friction and the road condition where the tires’ rolling on. 

In this study experimental data has been used to develop an estimator of the tire-road contact surface parameters based on strain data measurement. Firstly, the experimental data has been analyzed to derive relationships between the strain gauge measurements and the tire working conditions. To accomplish this analysis, herein has developed an algorithm in MATLAB able to make a selection of the key points of the strain measurements. The implementation of this algorithm allows a more accurate and standardized data selection reducing the margin of error in the relationships between the strain gauge and tire working conditions. Following on from the relationships derived from the analysis, a fuzzy logic system has been implemented to estimate the following tire parameters; slip angle, vertical and tangential forces in the contact patch. These results complement the conclusions of previous studies [16,17,18,19,20,21,22,23] to find the capacity of strain sensors to measure all the wheel forces.

As validation process, herein has been tested the outputs of the fuzzy logic estimator within complex maneuvers. The simulation software CarSim has been used to provide the data needed to accomplish this goal. Also, it has been used a semi-empirical model (Pacejka’s model) based on experimental data of the lateral force to compare the output of the fuzzy logic system at the same maneuvers.

One of the biggest features of this work has been to show the operational behaviour of intelligent tires as active sensors forming part of a vehicle. Further, it illustrates the information that may be provided by these sensors to ensure better dynamic performance of vehicles.

## 2. Strain-Based Intelligent Tire Systems

This section is about the process of strain data acquisition and collection of principal features. It will give the reader an overview of the experimental steps needed to develop this research, as well as, the data processing realized to pick out the main features. 

### 2.1. Experimental Strain-Based Intelligent Tire System

**Figure 1:**
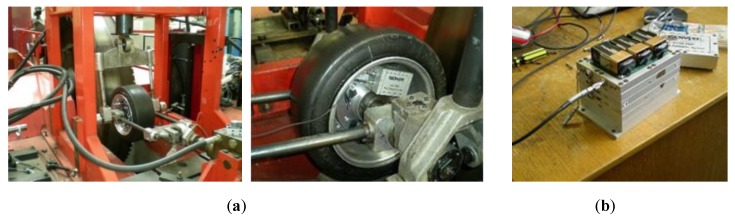
(**a**) Indoor tire test rig; (**b**) Acquisition system SoMat2000®.

Many research studies have chosen the strain sensor as the active element to provide information about the forces at the tire-road contact patch. Indeed, as explained in previous section, the tires as sensors can provide a strong relation with driving condition parameters, thereupon, through the deformation, it is possible to estimate tire parameters based on the strain [16,17,18,19,20,21,22,23,24]. This study is grounded on collected data that had been carried out in an indoor tri-axial tire test rig with strain-based intelligent tire system in the University of Birmingham Vehicle Dynamics Laboratory (see Figure 1). The indoor tire test rig allows variation in the speed, vertical load and slip angle. It also allows to measure the values of the longitudinal force for traction and braking rolling test and the lateral force for tire steady-state cornering rolling test [16,17,18,19,20,21,22,23,24]. 

The drum’s curved surface has a large diameter (2.44 m) so, its curvature has an insignificant effect on the results [20,25]. The indoor tire test rig is equipped with a drum speed controller and a signal conditioner to control the displacement and load. 

The intelligent tire prototype system is comprised of a test tire, the strain sensors, a SoMat2000® data acquisition system and a SoMat 2000 field computer. This data acquisition system is particularly suited for portable data collection.

The selected tire used was a DUNLOP SP SPORT 175/505 R13 (tubeless) slick radial tire. The test tire has been chosen taking into account the tire test rig limitations and the possibility to be used in an “FSAE” prototype as test car. Further stages of this study will be done in this “FSAE” prototype in order to extend the conclusions in real conditions, out of a laboratory tire test rig.

The experimental operational range of parameters implemented are defined according to the tire test rig limitation and the tested tire. The experimental operational range of parameters implemented are as follows:

- Tire inflation pressure: 0.8 bar–1.4 bar, step size: 0.2 bars.
- Tire preload: 250 N–1000 N, step size: 250 N.
- Tire speed: 10 km/h–50 km/h, step size: 10 km/h.
- Tire slip angle: 0°–10°, step size: 2°.
- Tire camber angle: 0° (due to the limitation in the test rig).

**Figure 2:**
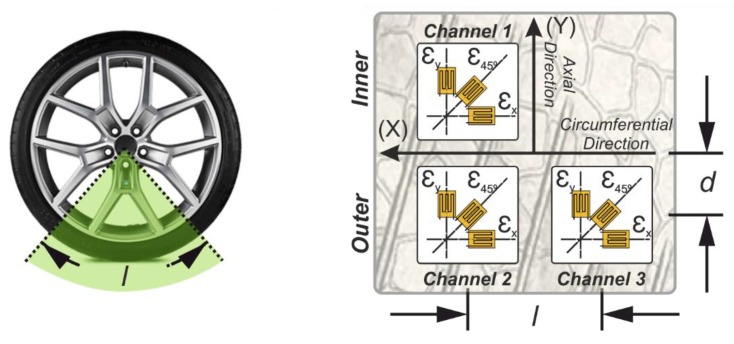
Multiaxial strain gauge disposition.

Three multiaxial strain gages (2 mm length—120 Ω gauge resistance) were set up, placed symmetrically at different points on the inner liner of the tire tread (see Figure 2). Two of them were placed in the same cross-section and the third one separated by 123.75 degrees of angular rotation. Figure 2 shows the strain gauges’ location scheme. The distance “d” and “l” are about 0.040 m and 0.515 m, respectively.

To measure the experimental data three channels were turned on at a sampling frequency of 1000 Hz, two of them for the axial strain measurement (${\mu \varepsilon }_{y1}$, ${\mu \varepsilon }_{y2}$—channels 1 and channel 3, respectively) located symmetrically with respect to tread center line and the other one to measure the circumferential strain (${\mu \varepsilon }_{x}$—channel 2). The channel 2 and 3, are located in the same cross section to measure the strains in the circumferential and axial directions when the tire is deflected by the influence of the lateral force. Additionally, this location provides interesting information due to the nonsymmetrical lateral behavior of the tire and certain external conditions such as camber angle [23].

The experimental measurements were collected by a set of data carried out at steady-state conditions for each tire operational condition. The measurement system was calibrated and zero error were checked before start the measurements of the system.

### 2.2. Selection of Characteristics for Measured Tire Strain

The data collected from experimental strain-based intelligent tire system is organized by the sets of data according to the slip angle, vertical force and tire rolling speed. Each set is formed by a time history of the strain measured at steady state condition. This order help to detail the effect of varying the tire operational conditions over the strain measurement. 

In Park et al. [26] it was pointed out that the distribution of contact stresses suffers variabilities due to changes of vertical load and inflation pressure. Under a constant tire load, the distribution of tire contact stresses are changed from higher contact stresses at the edge to higher stresses at the middle of the contact patch as tire inflation pressure increases. At the constant tire inflation pressure, the tire contact stresses at the edge area become higher as tire load increases. This information is of interest for the tire deformations measurements because they are carried out in three different points of the contact patch (see Figure 2, the strain multiaxial gauge disposition). Hence, in this work constant pressure under load variabilities has been considered to assure that the distribution of contact stresses is equal along the contact patch. Therefore, this work has considered the tire normal inflation pressure of 0.8 bar [24]. 

Figure 4a illustrates the set of data measured for channel 1 at the tire operational condition of 0.8 bar, 50 km/h and preloaded at 500 N. It can be noticed the repeatability in all the time history. Hence, an objective of this section is to automate the features extraction from the tire strain time history, being needed first to define the points of interest. According to Garcia-Pozuelo et al. [16,19,20], the most influential features to estimate tire parameters (e.g., vertical load, speed, and slip angle) are the tensile strain peaks and the offsets on the curve of strain in the time history, not considering the pressure effects despite the existence of a direct relationship between the pressure and the maximum compressive strain values, due to the increment in the stiffness of the tire [20]. For this purpose, this study focuses on the study of the tensile strain peaks in axial (lateral) and circumferential (longitudinal) directions, and the offset at each channel. 

**Figure 3:**
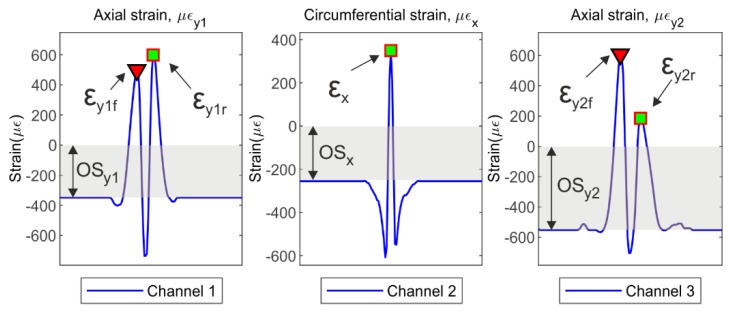
Features of the tire strain curve (0°, 0.8 bar, 500 N, 50 km/h).

In Figure 3 the most influential features (tensile strain peaks and the offsets) according to García-Pozuelo et al. [20] have been pointed out. These features are represented over the strain curves of the tire at channel 1, channel 2 and channel 3. The notation used for deformation features is as following, the axial strain offset, ${\mathrm{OS}}_{x}$, the axial strain offset, ${\mathrm{OS}}_{y2}$, the front/rear tensile axial strain peaks, $\varepsilon_{y1f}$/$\varepsilon_{y1r}$, the tensile circumferential strain peak, $\varepsilon_{x}$, and the front/rear tensile axial strain peaks, $\varepsilon_{y2f}$/$\varepsilon_{y2r}$. Finally, it is developed an algorithm capable of detecting the strain features into the strain curve at each operational condition. It automates the collection of the maximum tensile values for each channel and analyzes its variation for the different working conditions (speed, slip angle, and vertical load). 

**Figure 4:**
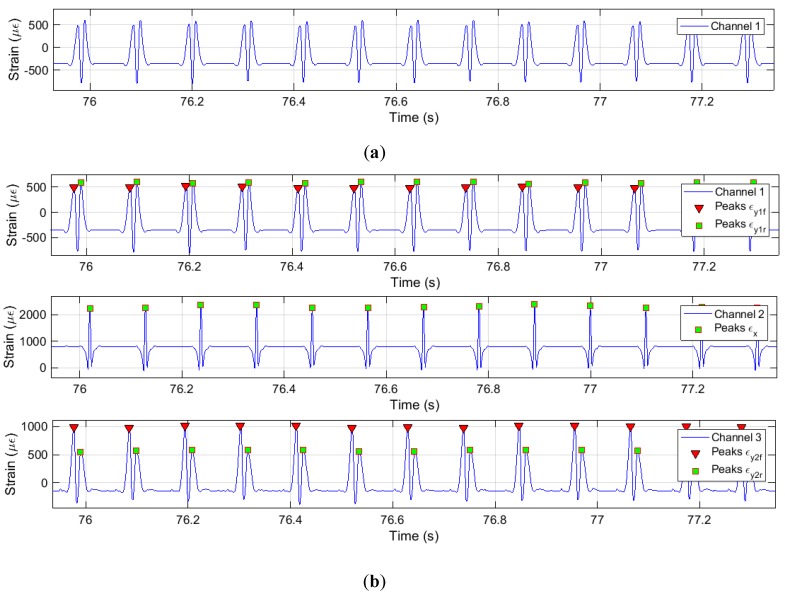
Intelligent Tire Strain time history at 0°, 0.8 bar, 500 N, 50 km/h. (**a**) Collected data of channel 1; (**b**) Detection peaks algorithm implemented.

Figure 4b illustrates the implementation of the algorithm for the tire strain time history at circumferential and axial directions. This algorithm is capable of detect the front and rear tensile peaks for the axial direction curves. Axial peaks are marked with the red triangle as front peak, and the green square as rear peak (see Figure 3). In order to analyze the collected data and achieve general conclusions the mean value for the set of representative peaks has been calculated (see Figure 4). The variation of these mean values for different tire conditions (vertical load, slip angle and rolling velocity) has been studied to propose robust estimation systems.

## 3. Strain-Based Method

In this work, the process of developing the strain-based method to detect tire parameters for Intelligent Tires is divided into four steps. The first and second steps are related to the Intelligent Tire Data Acquisition System and the data analysis, as previously described.

**Figure 5:**
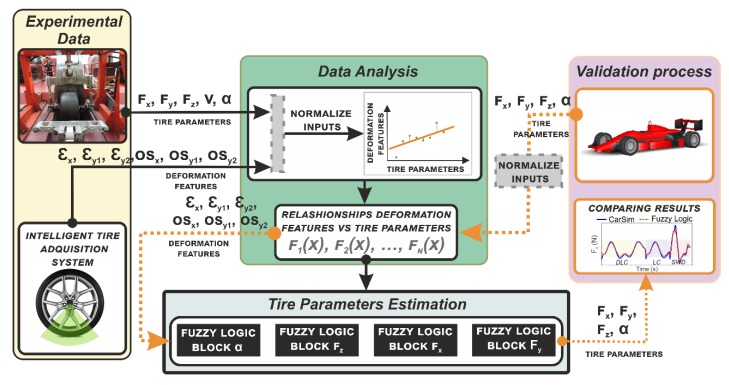
Working scheme used to develop the strain-based method.

In the third step, the Tire’s Parameter Estimation started with the fuzzy logic computational method, where, the available knowledge about the variations of the strain features is included in the fuzzy estimator by the formulation of the rules. Hence, the surface and curve fitting over the deformation features are the inputs whereas the values of the slip angle, radial load, and the tangential forces describe the outputs. Figure 5 shows the steps taken to develop the strain-based method proposed.

Once the proposed method is capable of detecting the tire parameters’ values, this work seeks to confirm the results to demonstrate the feasibility of the strain-based method. Therefore, a validation process is the fourth step (this process is indicated in Figure 5 with the dot arrows). It starts with the values of the tire parameters (lateral force, longitudinal force, vertical load and slip angle) described during a simulation maneuver.

In this case, the tire parameters were obtained from CarSim [27]. CarSim is a simulation software widely used in the automotive industry based on parametric modeling. The main advantage of using simulations software is the capacity to perform diverse types of vehicle maneuvers. The Formula 3 vehicle configuration (F3) was set up using experimental data. The maneuvers used to compare the results are Double Lane Change (DLC), Lane Change (LC), and Sine With Dwell (SWD) with speed of 30 km/h and 80 km/h on dry pavement.

The simulation parameters needs to be normalized to set up them into a range of [−1, 1]. According to the strain features relationships in the data analysis module, their inputs need to be into that bounds to then being turned them into deformation features (as it is depicted in Figure 5).

The next step is applying the Tire’s Parameter module (see Figure 5), where the resultant deformations are the inputs, and the outputs are the longitudinal, lateral and vertical forces and, the slip angle. Then a parallel display of the fuzzy logic estimations and the CarSim simulations is made to compare the results.

In order to back up the graphical evidence, provided by the validation process, about the effectiveness of the fuzzy logic estimation, the errors for different maneuvers, have been computed, picking as target output the simulations. According to Vargas-Meléndez et al, and Boada et al. [28,29], the normalized error as a function of time may be calculated by Equations (1)–(3), where, $y_{l}$ and ${\tilde{y}}_{l}$, are the simulation data and the fuzzy estimation results during a time period expressed as T, and the mean value $\mu_{l}$ of the target output:

(1) $$E_{t}=\;\frac{\varepsilon_{t}}{\sigma_{t}}$$

(2) $$\varepsilon_{t}{}^{2}=\;\int_{0}^{T}{\left(y_{l}-{\tilde{y}}_{l}\right)}^{2}\;dt$$

(3) $$\sigma_{t}{}^{2}=\;\int_{0}^{T}{\left(y_{l}-\mu_{l}\right)}^{2}\;dt$$

Furthermore, the experimental data has been used to develop an empirical model based on the so-called Magic Formula, chosen for its ability to produce characteristics that closely match measured curves for the side force [6,7,8,9]. For this case, it matches with the lateral force as a function of their respective wheel slip angle and vertical load. Afterward, this empirical model for describing lateral force behaviour has been used to compare with the proposed estimation.

### 3.1. Analysis of Operational Tire Parameters by Means of Experimental Tire Strains

**Figure 6:**
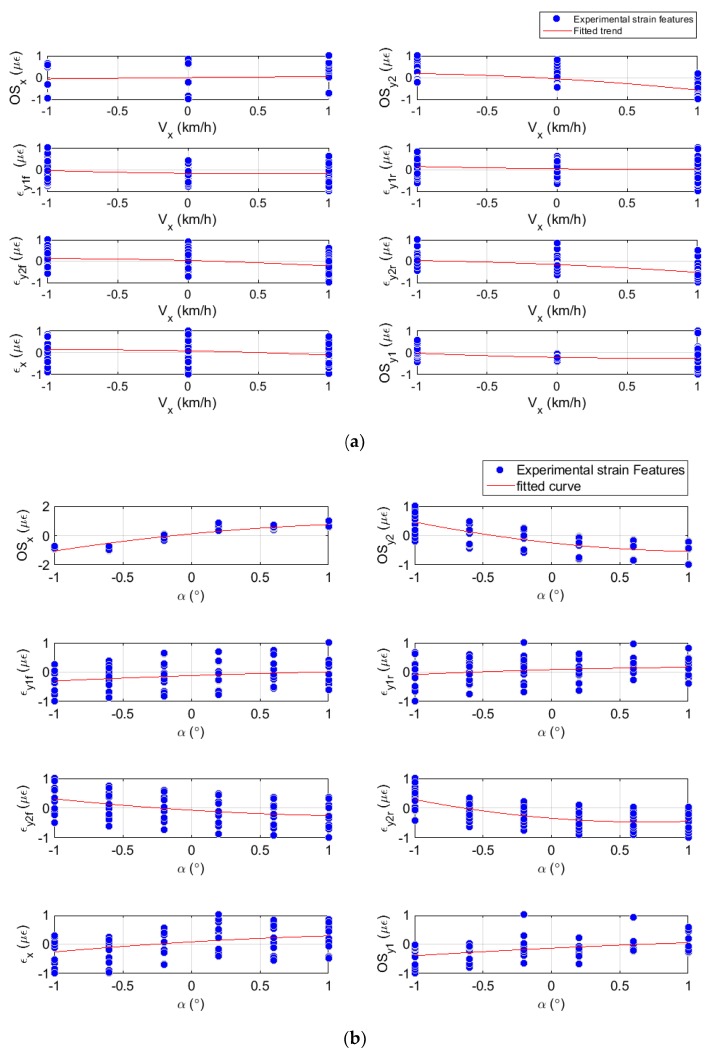
Simple curve fitting, variation of the strains features as function of potential parameters. (**a**) Strains feature as function of rolling speed. (**b**) Strains feature as function of slip angle.

**Figure 7:**
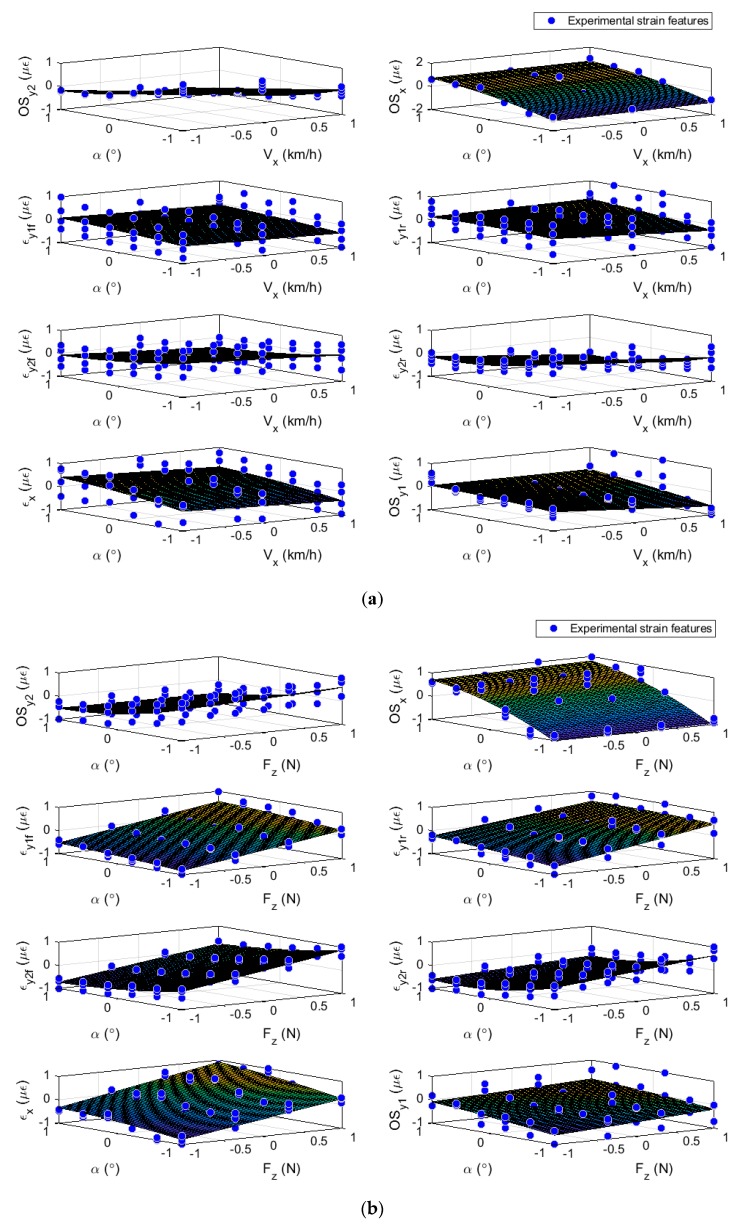
Curve surface fitting, variation of the strains features as function of potential parameters. (**a**) Strain features as function of slip angle and rolling speed. (**b**) Strain features as function of slip angle and vertical load.

Preliminary measurements of tire dynamic strain have been used to build relationships for developing the proposed estimation methodology. The tire strain features are regarded as the response variables and the tire working conditions as the input variables. Figure 6 and Figure 7 show the experimental data fitted by curves and by surfaces, where the trend of the strain features for different working conditions can be observed. In Figure 6 the blue dots show the experimental strain data at every specific condition and their trends are depicted by the red lines. In Figure 7 the fitted surfaces enable us to assess the strain features with two experimental conditions simultaneously. 

After identifying the pattern between the variables, it is possible to estimate a strain value at tire working conditions different from the experimental ones.

On the other hand, during the indoor experimental test the values of the lateral force, $f_{y}$, normal force, $f_{z}$, slip angle, α, rolling speed, V, and tire pressure, P, were collected, whereas, the longitudinal force value, $f_{x}$, was estimated according to Yang et al. [18]. There is established a relation between the tensile strain peaks ratio and longitudinal force values. Thus, the longitudinal force, $f_{x}$, for the experimental tests may be roughly equivalent to the results yielded by the relations claimed by Yang et al. [18,24].

As discussed above, this research is grounded on the Intelligent Tire experimental tests. It relates the strain features ($OS_{y2}$,$\;\varepsilon_{x}$, $\varepsilon_{y1f}$, $\varepsilon_{y1r}$, $\varepsilon_{y2f}$, $\varepsilon_{y2r}$,$\;OS_{x}$, $OS_{y1}$) to the tire working conditions, such as the slip angle, α, vertical force, $f_{z}$, lateral force,$\;f_{y}$, and longitudinal force,$\;f_{x}$. In this work the experimental data have been normalized in order to facilitate the data treatment for a deep analysis (see Figure 5). A range of data included between −1 and 1 can be analyzed in a more efficient way, providing robust relations not conditioned by its magnitudes. The same normalization method has been applied to the strain measurements and to the working conditions. First and second order polynomial functions were used for fitting the data. 

Figure 6 displays the relationship between the strain features and tire parameters, specifically the slip angle, α, and rolling speed, V. Figure 6a, shows that the speed influences the measurements in the axial direction (specifically in the outside part of the tire) and slightly in the circumferential direction. When the velocity increases (from 10 km/h to 50 km/h) it can be observed that the strain measurements at channel 3, tends to decreases; i.e., the offset, $OS_{y2}$, and strain peaks, $\varepsilon_{y2f}$ and $\varepsilon_{y2r}$. Moreover, in Figure 6b the strain measurement at channel 2 shows that the tensile values decrease and converge to a constant value as the slip angle increases from 0° to 10°, with step sizes of 2°.

Furthermore, the influence of the slip angle in the channel 2 (measurement in the circumferential direction) can be observed. The offset, $OS_{x}$, converge to a constant value and its low deviation makes it adequate to be used for slip angle estimation. 

Besides, comparing the trend of the offset of channel 2,$\;OS_{x}$, and the offset of channel 3,$\;OS_{y2}$, both strain gauges located in the outer part of the contact patch (see Figure 2), when the slip angle increases, the offset $OS_{x}$ also increases and the offset $OS_{y2}$ decreases. The variation in the channels located on the outer part are higher than the variation at the channel on the inner part. This fact makes the outer channels more sensitive and adequate to analyze the influence of the slip angle and the velocity changes at the contact tread. The fact that the tensile strain in the half of the contact patch of the tread band decreases agrees with the results of Yang et al. [17], “where the shape of the contact patch and the pressure distribution become asymmetric, such as, the lateral shear stress drastically varies on one side of the tire contact patch rather than the other side”. Also, the decreasing and increasing convergence of the axial and circumferential measurement might be attributed to inextensibility of the tire carcass, providing a real information about the tire parameters, e.g. lateral force, slip angle.

Similar plots have been produced for longitudinal force, lateral force and vertical load to note their influence over the strain features measurements. Owing to the high deviation that seems to be evident in most of the curve fitting, it has been considered to assess the variation of the deformation with more than one independent variable. 

Therefore, the use of surfaces is contemplated to provide a full picture from an overall perspective. To do this, two independents variables are considered with variables X and Y, as tire working conditions and a variable Z, as the mean of the strain parameters, where X, Y, and Z all fall onto a plane.

In Figure 6 and Figure 7 the experimental points of the strain features for different tire operational conditions have been plotted. Further, the curve fitting and the surface fitting were plotted, depending on the type of plot. Into the figures can be noted the trend of the strain features while the tire operational condition changes, independently of the specific value of this condition. In the surfaces’ representations, most of the strain features are structured in layers. The layers show the effect of the gap between the experimental tire operational conditions. As example, in Figure 7a the structured layers show the vertical load influence over the strain features graphic and in Figure 7b the velocity influence is shown. The information provided by the curves and surfaces, evidence the simultaneous influence of the operational conditions over the contact patch measurements on most of the strain features.

In Figure 7 the influence of the tire working condition on the strain features is clearer. The vertical load seems not to modify the offsets at any channel; nonetheless, the slip angle and the velocity influence them, more clearly at those channels in the outer part of the tread. The offset in the circumferential direction ($OS_{x}$) is slightly affected by the velocity, but the magnitude of the offset in the axial direction (at channel 2) decreases with the increment of the tire velocity (see Figure 7a,b). Examining the tensile strain features, the tensile strain values at channel 1 show a linear increasing trend due to the rise of vertical load independent of the slip angle, whereas, at channel 2, the increment is also linear but dependent on the slip angle and its magnitude decreased with increment in the velocity; in the circumferential direction the vertical load influenced the tensile strain magnitude ($\varepsilon_{x}$).

Figure 7a shows an example of surface fitting where the strain features $OS_{y2}$ and $OS_{x}$, are clearly functions of the tire operating parameters velocity and slip angle with a slight influence of the vertical load. It is evident that for these features ($OS_{y2}$, $OS_{x}$, and $\varepsilon_{y2r}$) which exhibit low deviation from the surface, the slip angle has a greater influence. However, contrasting Figure 6a and Figure 7a, the offset of the channel 2, $OS_{y2}$, is the only one which is notably affected by the velocity. 

This information suggests that the velocity has more effect on the outer part of the tire tread. In Figure 7b the tensile strain peaks of the channel 1 can be compared with the tensile strain peaks for channel 3. The plots for $\varepsilon_{y2f}$, $\varepsilon_{y2r}$, exhibit higher deviation from the surface than the peaks for channel 1. The slip angle clearly affects the offset at the outer part of the tire whereas the vertical load act upon the tensile features throughout the contact patch. 

The results shown in Figure 6b indicate a low dependence of the tensile peaks at channel 1 (axial strain) on the slip angle, whereas, the surface fitting (Figure 7b) clearly shows the goodness of fit of other strain features with the tire parameters. Further surface fittings showed similar results with most of the strain fits showing great precision. These findings are important for tire strain systems in order to standardize the variation of the strain features. The relationship between strain data and tire parameters (forces and slip angle) are expressed by the matrix [A]. This matrix includes a set of coefficients which characterize that relationship and it was built by using the study of the variations (see Equation (4)):

(4) $$\left[\begin{array}{c} \begin{array}{c} OS_{y2}\\ \varepsilon_{y2r} \end{array}\\ \vdots \\ \varepsilon_{y1r} \end{array}\right]=\left[A\right]\cdot \left[\begin{array}{c} \alpha \\ \begin{array}{c} f_{z}\\ f_{x}\\ f_{y} \end{array} \end{array}\right],$$

where the dependent variables can be the strain features ($OS_{y2}$,$\;\varepsilon_{x}$, $\varepsilon_{y1f}$, $\varepsilon_{y1r}$, $\varepsilon_{y2f}$, $\varepsilon_{y2r}$, $OS_{x}$, $OS_{y1}$) and, the working condition ($\;f_{x}$, $f_{y}$, $f_{z}$, α), can be defined as the independent variables. 

First of all, it is interesting to highlight that the apparent variability in the sets of data is due to different operational conditions are shown in each of them and don’t implies any repeatability issue. For instance, in Figure 7a the strain data are shown explicitly for different slip angles and velocities, but includes different vertical loads implicitly. This shows a certain stratification in the data for each condition. 

The information provided by these curves illustrate the influence of the rolling speed, the vertical load and the slip angle in the tire-road contact surface (two variables are shown explicitly and one of them implicitly). From the measurement can be deduced: the channels located in the outer part of the tire show more sensitivity to slip angle ($OS_{2y}$, $E_{2y}$ and $OS_{x}$) and the speed ($OS_{y2}$) variations. The vertical load has influenced both sides of the contact patch in a similar way. It should be taken into account that the experimental tests were carried out for steady state cornering conditions. In this condition, the tire rolling under the influence of the vertical load and the slip angle shows a lateral deflection distorting the shape of the contact patch and the strain distribution in it.

In previous works was shown the complexity of studying the tire through the variability found in the strain measurements under the simultaneous influence of operational conditions [1,13,16,19,20,21,22,23,30,31]. Herein has been simplified the analysis of the correlations of the experimental data to demonstrate that tire parameters and working conditions (lateral force, longitudinal force, vertical load and slip angle) can be estimated from the deformations measured in the contact surface with the strain-based Intelligent Tire system, extending the results from previous studies. Performing a deeper study of the experimental data to expand the analysis made and improve the estimator effectiveness is not ruled out.

### 3.2. Tire Parameters Estimation

This research started with investigating the variation of the strain values against the experimental conditions. In this phase, the possibility of fitting curves and surfaces of deformation variations has taken shape, being of great utility for applying a fuzzy logic estimator for longitudinal and lateral forces, vertical load, and slip angle. 

#### 3.2.1. Fuzzy Logic System Applied 

As discussed above, the Tire Parameter Estimation calls for the variations in the tire strain to characterize the tire deformation under certain operating conditions. The influence of tire operating parameters on the strain features is such that none of the single relationships gives by itself a reliable estimate of a tire parameter. Therefore, the use of fuzzy logic is adopted to correlate the features of the strain to give an accurate estimation of the tire parameter.

**Figure 8:**
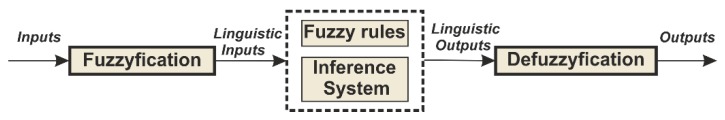
Processing steps of a fuzzy system.

Fuzzy Logic is a methodology that makes possible to estimate output values based on initial conditions known as inputs. A fuzzy logic system may divide into three steps as shown in Figure 8. According to Kiencke and Nielsen [32], the first step is fuzzification, where the inputs are converted into linguistic variables with memberships functions between 0 and 1. Afterwards, the linguistic inputs are then evaluated with an inference engine based on fuzzy rules and formed into fuzzy logic outputs. Lastly, the resulting fuzzy output is mapped to a crisp output using the membership functions in the step of defuzzification.

The defuzzification was carried out using the centroid method:

(5) $$y_{Def}=\;\frac{\int_{-\infty }^{+\infty }y\cdot \mu_{res}\left(y\right)\cdot dy}{\int_{-\infty }^{+\infty }\mu_{res}\left(y\right)\cdot dy},$$

where $\mu_{res}\left(y\right)$ is the aggregated membership function and $y_{Def}$ is the output variable. This work has been carried out using the Mamdani’s fuzzy inference and the triangular membership functions.

Based on previous studies of strain-based methods for Intelligent Tires [16,17,19,20], this study has enhanced the methodology used to estimate tire parameters. To improve the strain-based method requires using the slip and vertical load estimator to estimate the influence of tire operating parameters on tire strain features. Hence, the fuzzy logic system inputs work with normalized values of the fitted curves and surfaces. In Figure 5 the working scheme is shown, where the input and outputs of the fuzzy logic systems which are implemented into the Tire Parameters Estimation module can be noted. The normalization of data is also pointed out there. The normalization process has been done by mapping the minimum and maximum values of each feature to [$y_{min}$,$\;y_{max}$] based on the algorithm shown in Equation (6):

(6) $$y=\;\frac{\left(y_{max}-y_{min}\right)\cdot \left(x-x_{min}\right)}{\left(x_{max}-x_{min}\right)+y_{min}},$$

**Figure 9:**
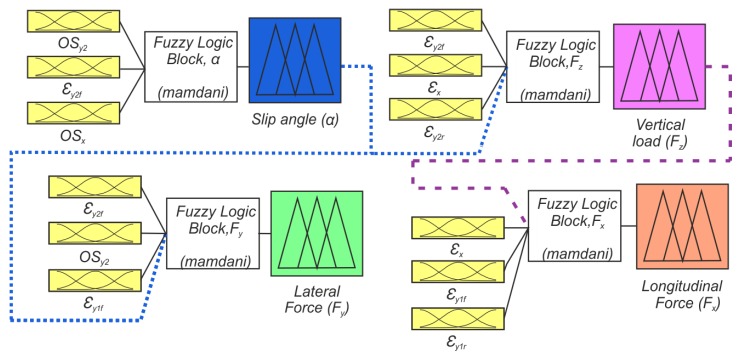
Architecture of the fuzzy logic system applied to develop the strain-based method.

The architecture of the fuzzy logic system applied to develop the proposed strain-based method is displayed in Figure 9. It is composed of four fuzzy logic blocks, each of them to estimate one tire parameter. Their inputs were chosen according to the impact of the tire working conditions on the tire deformation feature. The Tire Parameters Estimation phase in which the Fuzzy Logic Architecture detailed in Figure 9 is contained can be seen in Figure 5. The architecture of the fuzzy logic system starts by determining the slip angle values, followed by the vertical load, the lateral force and finally, the longitudinal force. Then the estimated slip angle values are fed into the vertical load and lateral force blocks (blue dot lines). Similarly, the vertical load value is one of the inputs for the longitudinal force block (purple dash lines). The continuous lines indicate deformation values. The fuzzy logic block to estimate the slip angle is defined by 21 rules and marking out as inputs: the offset of channel 3 (${\mathrm{OSy}}_{2}$), the tensile strain front peak of channel 3 ($\varepsilon_{y2f}$), and the offset of channel 2 ($OS_{x}$). These inputs are backed up by the curve fitting of the deformation features as a function of the slip angle. These strain features are shown to be more influenced by slip variation in Figure 6a,b. The selected inputs (${\mathrm{OSy}}_{2},\;\varepsilon_{y2f},\;OS_{x}$) are the same inputs previously used by Garcia-Pozuelo et al. [19] without using the rolling speed of the tire.

The fuzzy logic block for vertical load estimation has four inputs and 217 rules. The tensile peaks of channel 3 ($\varepsilon_{y2f}$ and $\varepsilon_{y2r}$) and channel 2 ($\varepsilon_{x}$), as well as, the slip angle estimated values are considered as inputs. Emphasizing the influence of the vertical load on the tensile strain peaks, the inputs of this block contemplate use of surfaces fitting as a function of the vertical load and slip angle to define the values of the selected strain features. 

Equally, fuzzy logic block to estimate the lateral force takes as inputs the values of tensile peaks channels 1 and 3 ($\varepsilon_{y1f}$ and $\varepsilon_{y2r}$) in addition to the offset values of channel 3 (${\mathrm{OS}}_{y2}$) and the slip angle. To generate deformation values, relationships of surfaces of $\varepsilon_{y2f}$, ${\mathrm{OS}}_{y2}$ (both as a function of lateral force and normal load) and $\varepsilon_{y1f}$ (as a function of lateral force and slip angle) are used. The final input is the estimated slip angle. This block uses a total of four inputs and 460 rules.

The final block is to estimate the longitudinal force with four inputs and a total of 145 rules. The inputs are the values of the tensile strain peaks of channel 2 ($\varepsilon_{x}$) and channel 1 ($\varepsilon_{y1f}$ and $\varepsilon_{y1r}$), in addition to the estimated vertical load values to provide a good estimation. Estimates of the deformation values are provided by a surface fit of the tensile strain peak ($\varepsilon_{x}$) as a function of the longitudinal force and vertical load, and curve fits of tensile strain peak values as a function of the longitudinal force. 

#### 3.2.2. Validating Fuzzy Logic System 

**Figure 10:**
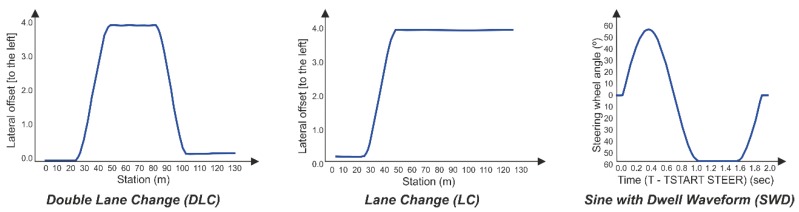
Lateral maneuvers.

In this work a strain-based methodology has been establishing to estimate tire parameters for Intelligent Tires. To check the fuzzy logic estimation system a validation process has been developed. This process has been identified by the orange dashed lines in Figure 5. It starts in the module of validation process where the values of the longitudinal force, lateral force, vertical force and slip angle are extracted from CarSim at severe maneuvers. These data are normalized and turned into deformation by the relationships of deformation features as function of tire parameters. Next, the strain features values are used to tests the fuzzy logic system in order to compare the results with CarSim. The maneuvers consisted of Double Lane Change (DLC), Lane Change (LC) and Sine With Dwell (SWD) as illustrated in Figure 10. Nonetheless, emphasizing the bounded range in the tests, the CarSim simulations at different maneuvers provide values out of the range, therefore, using the relationships of the normalized data the estimator is able to deliver the normalized outputs, and those outputs are compared with the original data from the maneuvers set in CarSim.

**Figure 11:**
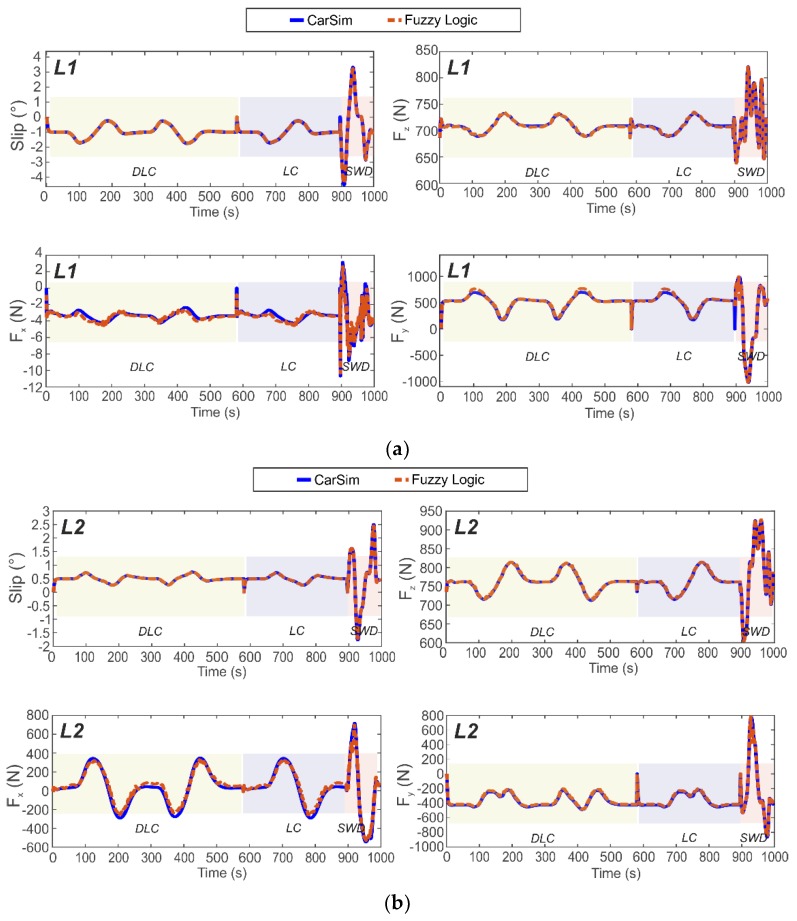
Simulation results for tire’s maneuverings at 30 km/h. (**a**) Steered axle, wheel L1 (**b**) Powered axle, wheel L2.

**Figure 12:**
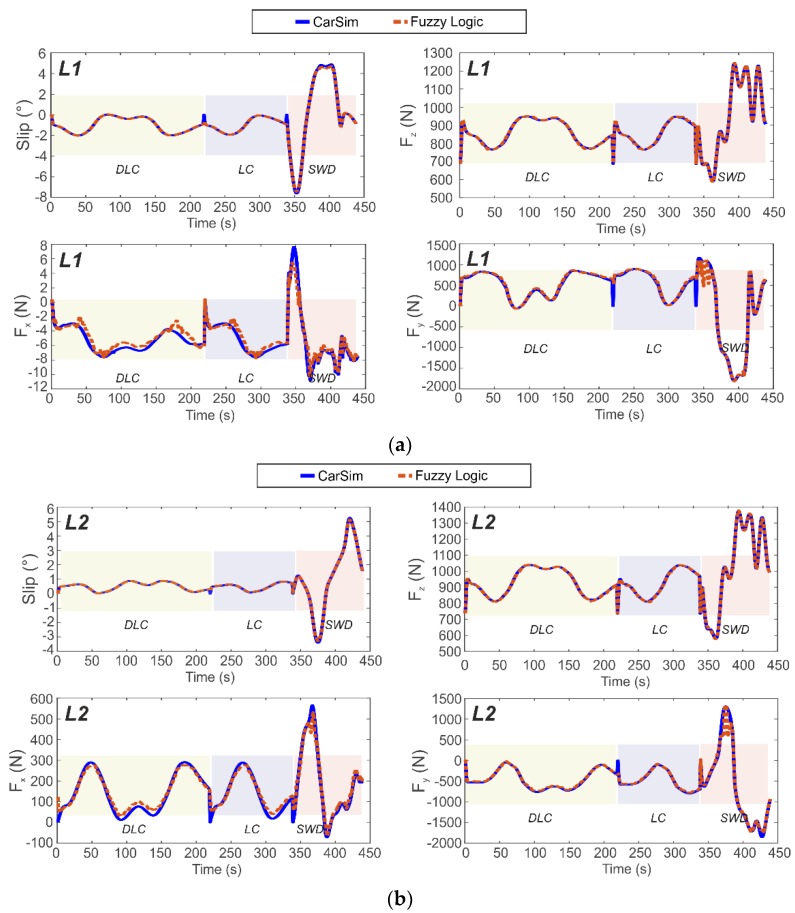
Simulation results for tire’s maneuverings at 80 km/h. (**a**) Steered axle, wheel L1 (**b**) Powered axle, wheel L2.

Figure 11 and Figure 12 illustrate the estimated results of the tangential forces at the contact patch ($F_{x}$ and $F_{y}$), vertical load ($F_{z}$) and slip angle (α) on the left side of the car (at left front wheel, L1, and left rear wheel, L2) for a combination of the three maneuvers. While the simulations were carried out at various velocities, the results displayed in Figure 11 and Figure 12 are for only 30 km/h and 80 km/h, respectively. The results obtained from tests carried out using the proposed estimator in these difficult lateral maneuvers show the good fidelity and sensitivity of the proposed approach. As a further illustration, the normalised errors of the simulations are presented in Table 1 for each wheel and velocity for three different velocities (30, 50 and 80 km/h).

An examination of Figure 11 and Figure 12 reveals that the proposed methodology to estimate tire parameter is highly accurate due to the excellent agreement between the simulation and the proposed estimation results. The vehicle configuration is rear traction wheel, L2. The proposed method is able to estimate the tire parameters at pure lateral condition or at combined slip situation, based solely on the information provided by the Intelligent Tire. Indeed, the relationships established from the strain gauge measurements enable us to describe the intelligent tire parameters for different operational conditions. 

The simulation results show that the proposed estimator is capable of predicting, with low margin of error, the target output in three severe maneuvers at 30, 50 and 80 km/h. This is also supported by the average errors indicated in Table 1.

Three velocities have been shown in Table 1: one in the middle of the experimental test values, other on the boundary of the test values and the last one, outside the bounds of the real test in order to illustrate one of the advantages found when using the normalization to feed the fuzzy logic algorithm. The curves for different operational conditions (vertical load and rolling speed) in the surface’s representations seem to be morphologically similar.

These figures demonstrate that the most difficult maneuver for accurately estimating the tangential forces is the Sine With Dwell (SWD) maneuver, whereas for slip angle and vertical load the prediction shows good results. Despite this, in the majority of cases the proposed estimator accurately predicts the target output.

The differences between the estimated and the simulated longitudinal force may be attributed to inaccuracies in the experimental longitudinal force values which are estimated rather than measured as indicated above. It is also evident from their error values, which are higher than the rest. Despite this, it has been shown that they are not unduly affected by the assumptions used in their estimation.

It can be observed that the values of normalized errors calculated for the proposed estimator are all less than one. It means that the estimation results are acceptable since the root mean square error between the estimated results and the reference output (CarSim simulations) are within the deviation allowed for the reference output. 

#### 3.2.3. Modeling of the Lateral Force

To describe the interaction between tires and road surface is of vital importance for the study of lateral vehicle motion due to the influence of the tire-road contact forces on vehicle dynamics behaviour. This section focuses on describing the lateral force in a combined slip situation with a mathematical model to test the estimation results with the experimental data. 

Additionally, the achieved results by means of the fuzzy logic estimator have been compared to a semi-empirical model proposed by Rajamani, Bakker et al. [9] and Pacejka et al. [6,7,8]. The lateral force value collected at tire test rig for different tire operational condition has been used to fit the semi-empirical model.

The general form of the Magic Formula, derived from experimental data, is shown in Equations (7) and (8), where the variable Y is the output variable $F_{y}$, and x is the slip angle as an input variable. The coefficients B, C, D, E, $S_{v}$, $S_{h}$, take account of the camber angle, the cornering stiffness and the load variations [9]. To determine their values, MATLAB toolbox of curve fitting is applied to measured data to obtain the best fit. The Magic Formula parameters obtained from the lateral force experimental data are indicated in Table 2. The experimental data was obtained for various values of the vertical load for a zero camber angle, picking it as another input variable:

(7) $$y=D\cdot \sin\left[C\cdot \tan^{-1}\left\{Bx-E\left(Bx-\tan^{-1}Bx\right)\right\}\right]$$

with:

(8) $$Y\left(x\right)=y\left(x\right)+S_{v}\;x=X+S_{h}$$

**Figure 13:**
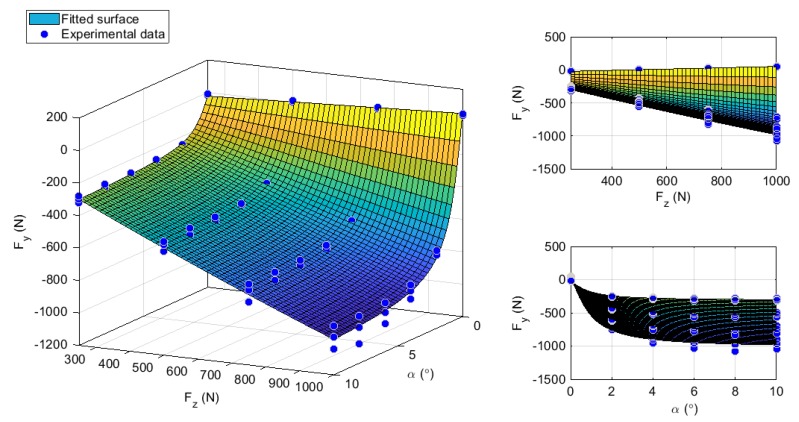
Experimental lateral force fitted surface based on Pacejka model at 30 km/h.

As is shown in Figure 13, the experimental data produces a curve that passes through the origin of the axes of the lateral force and slip angle. It may be observed that the lateral force reaches a maximum value and subsequently tends to a horizontal asymptote through variation of slip angle. Bringing the influence of the vertical force out, the lateral force increases with the increment of vertical load. Consequently the vertical load has been integrated into the model by the coefficients D that represents the peak value and by the vertical shift, $S_{v}$. The coefficients, D and $S_{v}$, are replaced by the relation (see Equations (10) and (11)) suggested by Rajamani et al. [9] and Pacejka et al. [6,7,8]. Figure 13 highlights the shift due to the vertical force, which might be due to the ply steer, conicity and rolling resistance explained by Bakker et al. [6]. Although the stiffness factor B and curvature factor E are functions of the vertical load, they can be directly defined by the surface fitted data. Equation (9) shows an adaptation of Equation (7) to include vertical force variation as another input value where a, b, c, d, B, C, E, are constant coefficients for the fitted data:

(9) $$F_{y}=(F_{z}\cdot \left(a\cdot F_{z}\right)+b)\cdot \sin\left[C\cdot \tan^{-1}\left\{B\cdot x-E\left(B\cdot x-\tan^{-1}\left(B\cdot x\right)\right)\right\}\right]+c\cdot F_{z}+d,$$

(10) $$D=\;F_{z}\cdot \left(a\cdot F_{z}\right)+b,$$

(11) $$S_{v}=\;c\cdot F_{z}+d$$

Further, Figure 13 illustrates the influence of velocity by splitting the experimental points as the vertical load and slip angle are increased. The comparison has been made at 30 km/h.

**Figure 14:**
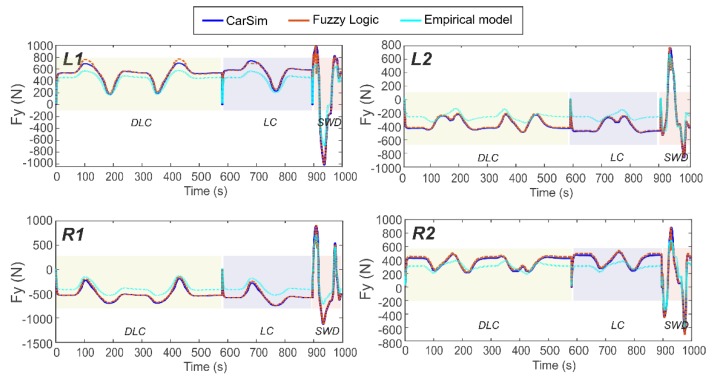
Comparison of the lateral force estimated by the experimental models, estimated by Fuzzy Logic and CarSim simulation at 30 km/h.

In the following, the model has been tested and compared with the simulation result for the same vehicle maneuvers and compared with the proposed estimator results. Table 3 displays the errors estimated for the experimental model and the fuzzy logic estimation in comparison with the simulated values. The complete dataset made up of the linear and non-linear dynamic conditions (severe maneuvers) shows that the proposed estimation model, Pacejka’s model, presents certain differences with fuzzy logic (see Figure 14). The most important result is that under these conditions, it can be seen that the peaks of both curves show similar values and these are the most representative points in the range of severe maneuvers. It can also be seen that the curves show better similarities for the front tires, with higher slip angles, than for the rear tires. The differences in the values away from these peaks are less significant than in these key points. These differences might be due to inaccurate coefficients in the model or due to the limitations of Pacejka’s model when it is used to predict combined high demanding conditions. This will be analyzed more deeply in further studies.

To support the graphical evidence, the normalised error is supplied in all cases (see Table 3), in comparison with the target output of the simulation. Figure 14 shows that the proposed estimation (fuzzy logic) is able to estimate the lateral force more accurately. Parallels between the proposed empirical model, the estimation provided by fuzzy logic and the CarSim result can be noted. The parallels of these results is also demonstrated with the computed normalised error values, for the proposed estimation it varies between 0.112–0.156 and for the empirical model it varies between 0.316–0.816, depending on the tire position. 

It is evident that the fuzzy logic estimation accurately represented the lateral force at each tire during slip angle variations (see Figure 11a). Since the empirical model is considered less accurate than the experimental results, it is assumed that some improvement of the proposed empirical model is needed, but this is not the aim of this study. As discussed above, the present work is not focused on the use of semi-empirical Pacejka’s model. At the contrary, the main aim is to develop a methodology to estimate the tire parameters by means of fuzzy logic algorithms. Pacejka’s model is just used in order to compare the results of the estimations with other methodology (a consolidated tire model fitted from experimental data).

The resulting normalised errors are displayed in Table 3. In all cases, the proposed estimator shows a better performance and with considerably smaller errors than the empirical model. It is important to note the influence of the slip angle over the empirical model, i.e. that at large values of slip angle its prediction is better.

## 4. Discussion

**Figure A1:**
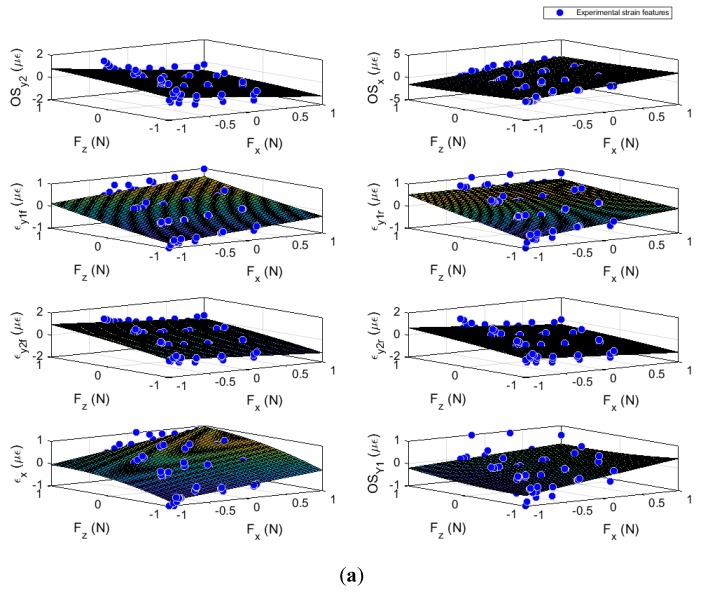

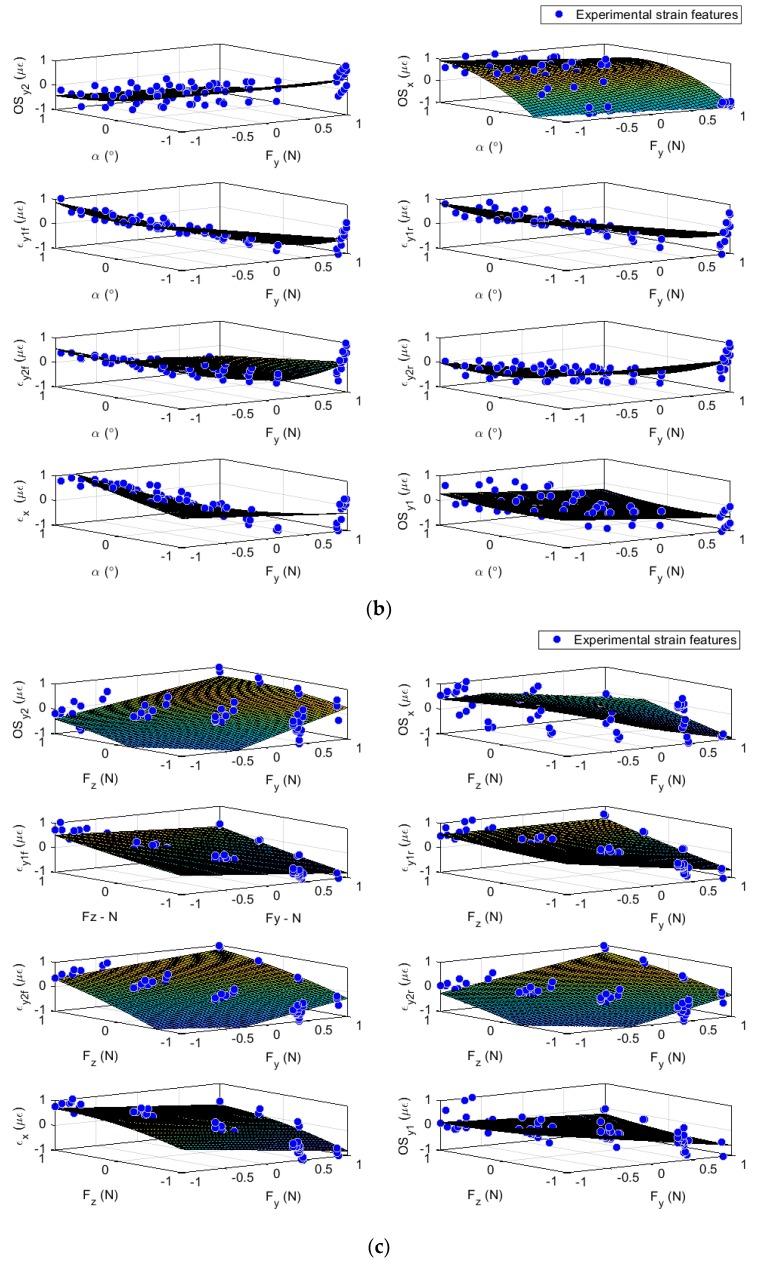
Curve surface fitting, variation of the strains features as function of potential parameters. (**a**) Strain features as function of vertical load and longitudinal force. (**b**) Strain features as function of slip angle and lateral force. (**c**) Strain features as function of vertical load and lateral force.

Figure 7a,b shows the influence of the tire parameters on the strain features. Surfaces were formed to display the variation of the strain features resulting from varying of vertical load and rolling velocity. In some cases, the surface fit seems to describe the majority of points (see also Figure A1 in the Appendix A), although for further studies implementation of a deeper analysis of the experimental data is proposed. The use of normalized correlations between the operational condition and the strain gauges measurements allows us to work directly with the trends (avoiding the use of specific experimental points for every estimation). Therefore, the fuzzy logic system is managed by the integration of these trends yielding accurate results. Further, the previous normalization of the data has it made possible to estimate a normalized output and, therefore, estimate tire conditions out of the bounds of the experimental range. As result, the strains collected by the intelligent tire in the contact patch area are used to determine the values of tire parameters for severe maneuvers conditions through the methodology proposed. Figure 11 and Figure 12 show the results yielded by the proposed methodology, where a good fidelity at both the 30 and 80 km/h results is noted.

In Section 3.2.2, the ability of the proposed empirical model to predict the target output is better for a pure slip condition than for combined situations, where the empirical model seems more affected. According to Rajamani [9], analytical models do not always lead to quantitatively accurate results. Differences from experimental data were observed, especially at large slip and at combined slip. This may be happening at steered wheel, L1, and at powered wheel, L2, where their values of vertical force are similar, nonetheless, results for the traction wheel were less accurate using the empirical model (see Figure 11a,b and Figure 14). Something similar happened for the right side of the vehicle (R1 and R2), where the estimation of a combined situation was less accurate.

## 5. Conclusions

This paper has investigated the detection of the lateral force, longitudinal force, vertical load and the slip angle through strain-based Intelligent Tires using fuzzy logic estimation. The proposed method has made use of curve and surface fittings of the deformation features and normalized the data to correlate the variations between them, thus yielding acceptable estimation for the slip angle and the tire-road contact forces, founded on graphical and numerical evidences. The knowledge about the variations of the tire deformation in the contact patch has been helpful for accomplishing the estimation of all forces. Moreover, it is necessary to highlight the behavior of the tire’s strains experimental data at different positions in the tread band (inner and outer parts of the contact patch), being possible to identify that the influence of the velocity and the slip angle are more clear and significant on the outer part of the tread band. 

One of the limitations of this kind of study is being able to measure the behaviour of an Intelligent Tire estimator for real car maneuvers. Nonetheless, in this work, it has been possible to assess the behaviour of the proposed method to estimate the tire-road contact forces in complex maneuvering by means of simulation tools. An FSAE prototype is being adapted as test car for further stages of this study in order to extend the conclusions in real conditions, out of a laboratory tire test rig. Looking at the estimations for inputs outside the range of explicit experimental points, it is observed that the process of normalization provides to the fuzzy logic system the capacity to estimate suitable results. 

It also demonstrates that deformation of the tire contact patch is highly correlated with the tire parameters emphasizing the essential role of the tire as a vehicle sensor. This combination also is a good approach in order to reduce the number of tests needed. 

Furthermore, the proposed method is able to estimate the tire parameters under pure lateral conditions or for combined slip situations, based only on the information provided by the Intelligent Tire, while the empirical tire model has limitations, as has been shown. 

The values of normalised error achieved by the proposed estimator for tire road parameters demonstrates the effectiveness of strain-based Method for Intelligent Tires. Further, the ability of the strain-based Intelligent Tire to estimate the tire parameters of each tire through contact patch may be significantly useful for vehicle dynamic behaviour offering the possibility to predict the load transfer or friction coefficient. A whole set of working condition combinations has been taken into account in the design of experiment in order to carry out the test [19,20]. However, a deeper study of the experimental data could yield a more precise estimator. Additionally, in this research only the experimental data of a tire slick radial tire DUNLOP SP SPORT 175/505 R13 (tubeless) has been used to develop a strain-based Intelligent Tire. It would be interesting to implement this study with other types of tire to compare the differences that might exist between them. With this aim an Avon 175/53R13 slick tire has been instrumented to complement these conclusions and the results will be reported in due course.

## Figures and Tables

**Table 1:** Normalized error of tire parameters simulation results.

Velocity (km/h)	Tire	α	$F_{z}\;(-)$	$F_{x}\;(-)$	$F_{y}\;(-)$
30	L1	0.049	0.063	0.346	0.156
	R1	0.023	0.039	0.419	0.117
	L2	0.023	0.039	0.177	0.121
	R2	0.027	0.037	0.233	0.149
50	L1	0.022	0.036	0.361	0.141
	R1	0.021	0.036	0.337	0.151
	L2	0.024	0.026	0.152	0.138
	R2	0.025	0.025	0.229	0.146
80	L1	0.041	0.026	0.274	0.119
	R1	0.026	0.032	0.256	0.155
	L2	0.026	0.025	0.182	0.142
	R2	0.024	0.025	0.201	0.112

**Table 2:** Parameters for empirical model of lateral force fitting and goodness of fit.

Coefficients		Confidence Bounds at 95%	Goodness of Fit
B	0.142	(−4.771; 5.056)	SSE: 7.699e+04 R-square: 0.990 Adjusted R-square: 0.989 RMSE: 34.416
E	0.121	(−4.258; 4.499)	
C	0.093	(−66.100; 66.290)	
a	7.435e-04	(−0.524; 0.526)	
b	−8.444	(−5973; 5956)	
c	0.099	(0.045; 0.154)	
d	−47.922	(−82.330; −13.510)	

**Table 3:** Contrasting normalised error of tire lateral force.

Velocity (km/h)	Tire	$F_{y}$ Fuzzy Logic (-)	$F_{y}$ Pacejka (-)
30	L1	0.156	0.429
	R1	0.117	0.646
	L2	0.121	0.816
	R2	0.149	0.600
50	L1	0.141	0.316
	R1	0.151	0.471
	L2	0.138	0.567
	R2	0.146	0.402
80	L1	0.119	0.318
	R1	0.155	0.405
	L2	0.142	0.489
	R2	0.112	0.323

## Appendix A

This appendix contains the rest of the relevant surfaces to show the reader how almost all the experimental points may approximate a surface fit. This research was based on the use of this type of fit to approximate deformation values.
